# Endurance exercise resistance to lipotoxic cardiomyopathy is associated with cardiac NAD^+^/dSIR2/*PGC-1α* pathway activation in old *Drosophila*

**DOI:** 10.1242/bio.044719

**Published:** 2019-10-15

**Authors:** Deng-Tai Wen, Lan Zheng, Jin-xiu Li, Dan Cheng, Yang Liu, Kai Lu, Wen-qi Hou

**Affiliations:** 1 Key Laboratory of Physical Fitness and Exercise Rehabilitation of Hunan Province, Hunan Normal University, Changsha 410012, Hunan Province, China; 2 Department of Sports Science, Ludong University, Yantai 264025, Shandong Province, China

**Keywords:** Heart, *dSir2*, NAD^+^, *PGC-1α*, Exercise, High-fat diet

## Abstract

Lipotoxic cardiomyopathy is caused by excessive lipid accumulation in myocardial cells and it is a form of cardiac dysfunction. Cardiac *PGC-1α* overexpression prevents lipotoxic cardiomyopathy induced by a high-fat diet (HFD). The level of NAD^+^ and *Sir2* expression upregulate the transcriptional activity of PGC-1α. Exercise improves cardiac NAD^+^ level and PGC-1α activity. However, the relationship between exercise, NAD^+^/dSIR2/*PGC-1α* pathway and lipotoxic cardiomyopathy remains unknown. In this study, flies were fed a HFD and exercised. The heart *dSir2* gene was specifically expressed or knocked down by UAS/hand-Gal4 system. The results showed that either a HFD or *dSir2* knockdown remarkably increased cardiac TG level and *dFAS* expression, reduced heart fractional shortening and diastolic diameter, increased arrhythmia index, and decreased heart NAD^+^ level, dSIR2 protein, *dSir2* and *PGC-1α* expression levels. Contrarily, either exercise or *dSir2* overexpression remarkably reduced heart TG level, *dFAS* expression and arrhythmia index, and notably increased heart fractional shortening, diastolic diameter, NAD^+^ level, dSIR2 level, and heart *dSir2* and *PGC-1α* expression. Therefore, we declared that exercise training could improve lipotoxic cardiomyopathy induced by a HFD or cardiac *dSir2* knockdown in old *Drosophila*. The NAD^+^/dSIR2/*PGC-1α* pathway activation was an important molecular mechanism of exercise resistance against lipotoxic cardiomyopathy.

## Abstract (precis)

**Summary:** Exercise training prevents lipotoxic cardiomyopathy induced by a high-fat diet or cardiac dSir2 knockdown in old *Drosophila*.

## INTRODUCTION

Cardiac disease is a major cause of mortality in modern society. The high prevalence of obesity and related diseases, such as lipotoxic cardiomyopathy, plays a significant role in the increased incidence of heart failure. Heart failure affects more than a billion people worldwide. Lipotoxic cardiomyopathy is caused by excessive lipid accumulation in myocardial cells, and it is a form of cardiac dysfunction (Borradaile and Schaffer, 2005). For example, high-fat diet (HFD)-fed flies exhibit increased triglyceride (TG) fat and alterations in insulin/glucose homeostasis, similar to mammalian responses. A HFD also causes cardiac lipid accumulation, cardiac contractility reduction, conduction blocks and severe structural pathologies, reminiscent of diabetic cardiomyopathies (Birse et al., 2010). In addition, increasing evidence shows that inhibition of signaling through insulin, target of rapamycin (TOR), or the lipogenic transcription factor SREBP, or by increasing TG lipolysis, is effective in counteracting excess lipid accumulation as well as the associated cardiac defects in flies (Birse et al., 2010; de Paula et al., 2016; Hardy et al., 2015). These reports suggest that both a HFD and cardiac lipid metabolism genes are closely related to lipotoxic cardiomyopathy.

*Sir2* is the most intensively discussed longevity gene in current aging research. Importantly, some studies have shown that *Sir2* is involved in lipid metabolism regulation; a screen for obesity-inducing genes in *Drosophila* larvae pointed to a role for *Sir2* in regulating fat metabolism and a response to amino-acid starvation (Reis et al., 2010). Moreover, *Sir2* apparently regulates expression of genes involved in fat metabolism, and the lack of *Sir2* increases fat deposition under normal conditions and consequently impairs starvation survival of flies (Banerjee et al., 2012). Next, a recent study shows HFD-fed flies exhibit increased body TG levels and decreased body *dSir2* expression (Wen et al., 2018). However, it is unclear whether *Sir2* can take part into heart lipid metabolism regulation. A recent study has confirmed *PGC-1α* as a vital antagonist of HFD-induced lipotoxic cardiomyopathy in flies since it plays key roles in mitochondrial biogenesis and electron transport chain assembly (Diop et al., 2015; Dumont et al., 2018). Interestingly, *Sir2* expression can alter the transcriptional activity of the mitochondrial biogenesis coactivator PGC-1α, and it catalyzes PGC-1α deacetylation both *in vitro* and *in vivo*. Overexpression of Sir2 deacetylase or increasing NAD^+^ levels activates transcriptional activity of PGC-1α in neurons and increases mitochondrial density (Dabrowska et al., 2016; Lan et al., 2017). Therefore, studying the relationship between cardiac NAD^+^/dSIR2/*PGC-1α* pathway and heart lipid metabolism is very important to understand the mechanism of lipotoxic cardiomyopathy formation.

In modern society, exercise combined with a healthy diet is considered the most economical and non-invasive way to prevent and treat obesity (Bales and Porter Starr, 2018). Exercise also improves heart function and decreases incidence of heart failure in both human and *Drosophila*. Recent studies report that endurance exercise improves cardiac contraction, and it reduces body and heart lipid levels and heart fibrillation in both HFD and aging flies (Wen et al., 2018; Zheng et al., 2017). Similarly, exercise training alters extrinsic modulation of the heart and improves the intrinsic pump capacity of the heart in human, and it also improves quality of life of patients with chronic heart failure (Chrysohoou et al., 2014; Jackson, 2000; “Water exercise safe for troubled hearts”, 2011). In addition, exercise increased muscle NAD^+^ levels (Green et al., 1992; Koltai et al., 2010) and increasing NAD^+^ levels activates transcriptional activity of PGC-1α in neurons and increases mitochondrial density (Dabrowska et al., 2016; Lan et al., 2017). Therefore, these results suggest that NAD^+^/dSIR2/*PGC-1α* pathway activation may be one of the key mechanisms in which exercise improves heart function and prevents lipotoxic cardiomyopathy.

In this study, to explore whether endurance exercise could resist HFD-induced lipotoxic cardiomyopathy via activating NAD^+^/dSIR2/*PGC-1α* signal pathway, experimental flies were fed a HFD and given exercise, and heart *dSir2* expression was changed by building UAS/hand-Gal4 system. The heart TG levels and *dFAS* gene expression were reflected in the lipid metabolism status. In addition, the heart diastolic diameter, systolic diameter, fractional shortening and arrhythmia index were reflected in the heart function. Finally, the cardiac NAD^+^ levels, dSIR2 protein level, *dSir2* mRNA expression and *PGC-1α* mRNA expression were reflected in the heart NAD^+^/dSIR2/ *PGC-1α* pathway status.

## RESULTS

### Exercise prevented lipotoxic cardiomyopathy and activated cardiac NAD^+^/dSIR2/*PGC-1α* pathway in *Drosophila*

Increasing evidence confirms that heart lipotoxicity impairment can be induced by feeding HFD in both mammals and flies, and the heart contractility and ejection fraction were weakened at the same time (Abdurrachim et al., 2014; Sibouakaz et al., 2016). Lipotoxicity impairment is also accompanied by the dysfunction of some genes in the heart, such as *PGC-1α*, *dFAS*, *TOR*, etc. (Birse et al., 2010; de Paula et al., 2016; Diop et al., 2015). On the contrary, exercise training improves heart function, decreases incidence of heart failure in both mammals and *Drosophila*, and reduces body and heart fat levels and heart fibrillation (Wen et al., 2018; Zheng et al., 2017). In addition, exercise increased muscle NAD^+^ levels (Green et al., 1992; Koltai et al., 2010), and the increasing NAD^+^ levels could activate transcriptional activity of PGC-1α in cells and increase mitochondrial density (Dabrowska et al., 2016; Lan et al., 2017). These results hinted that NAD^+^/dSIR2/*PGC-1α* pathway activation may be one of key mechanisms that exercise improved heart function and prevented lipotoxic cardiomyopathy. To identify this hypothesis, fruit flies in this experiment were subjected to exercise intervention and a HFD intervention.

**Fig. 1.**
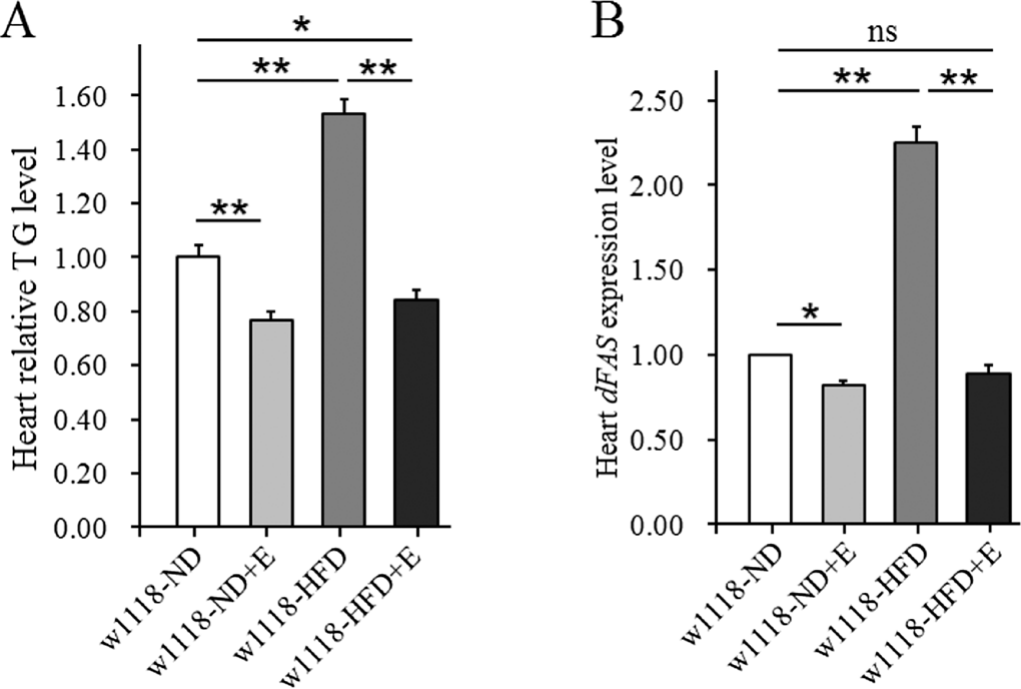
**The effect of a HFD and exercise on heart lipid accumulation.** (A) Heart relative TG level. Results are expressed as the fold difference compared with *w^1118^*-ND flies. The sample size was 80 hearts with three biological replicates. (B) Heart *dFAS* expression level. The sample size was 80 hearts with three biological replicates. A two-way ANOVA was used to identify differences among the ND, ND+E, HFD, and HFD+E groups in *w^1118^* flies. Data are represented as means±s.e.m. *P<0.05; **P<0.01.

In this study, our results showed that a HFD remarkably increased heart TG levels in untrained-*w^1118^* flies (*P*<0.01), and it also upregulated heart *dFAS* expression levels (*P*<0.01). These were consistent with the results of previous studies. In addition, we found that exercise availably reduced heart TG level and *dFAS* expression level in both *w^1118^*-normal diet (ND) and *w^1118^*-HFD flies (*P*<0.01, *P*<0.05). Interestingly, the heart TG levels in *w^1118^*-high-fat diet+exercise (HFD+E) flies were lower than those in *w^1118^*-ND flies (*P*<0.05). Therefore, we identified that endurance exercise could prevent lipid accumulation by downregulating cardiac *dFAS* expression level (Fig. 1A,B).

**Fig. 2.**
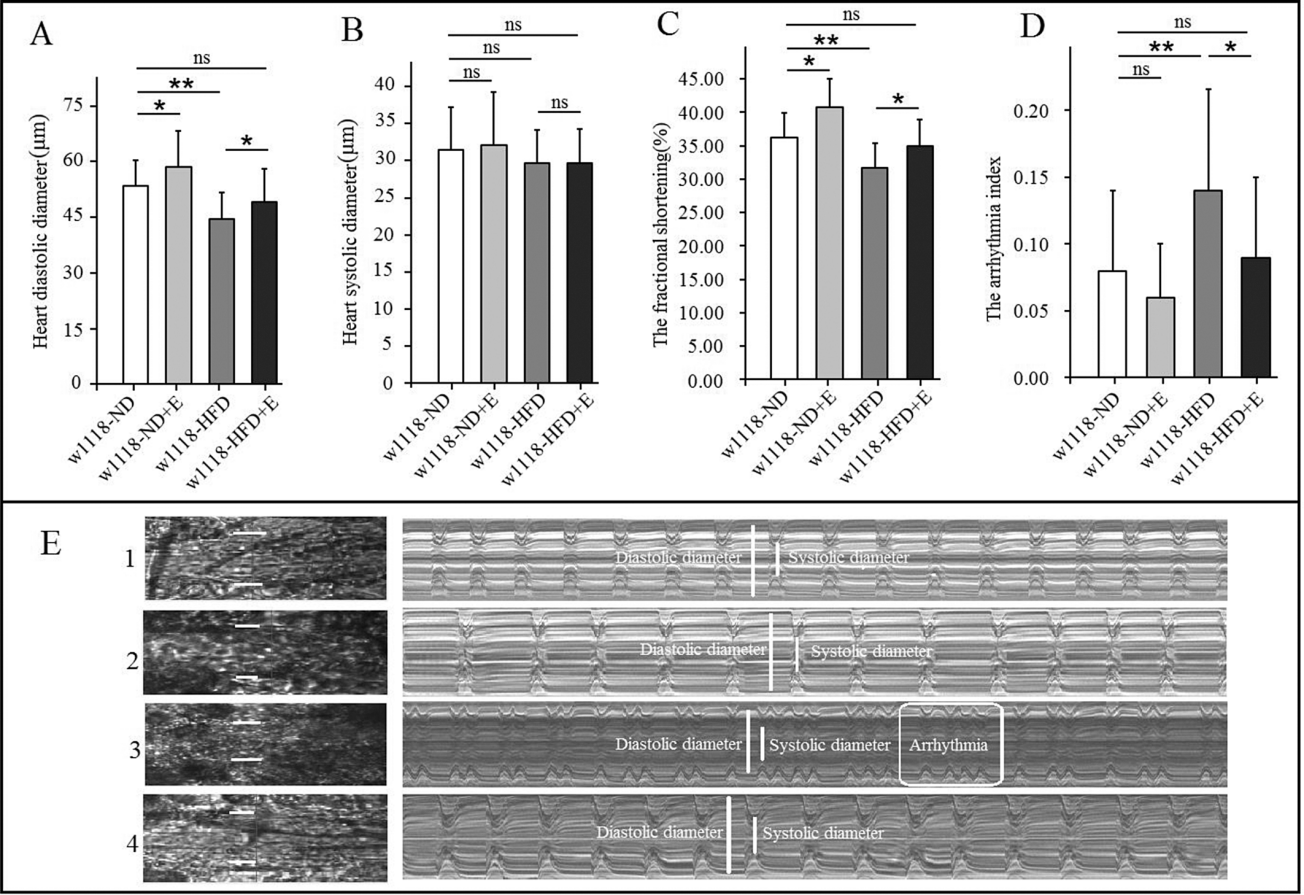
**The effect of a HFD and exercise on heart function.** (A) Heart diastolic diameters. (B) Heart systolic diameters. (C) Fractional shortening. (D) Arrhythmia index. (E) Illustrating qualitative differences in heart function parameters (10 s): fractional shortening and arrhythmia index; E1: *w^1118^*-ND; E2: *w^1118^*-ND+E; E3: *w^1118^*-HFD; E4: *w^1118^*-HFD+E. A two-way ANOVA was used to identify differences among the ND, ND+E, HFD, and HFD+E groups in *w^1118^* flies. Data are represented as means±s.e.m. *P<0.05; **P<0.01. The sample size was 30 hearts.

For heart function, results displayed that a HFD significantly reduced heart fractional shortening (FS) in untrained *w^1118^* flies (*P*<0.01), and it also notably decreased heart diastolic diameters in untrained *w^1118^* flies (*P*<0.05). Exercise significantly increased FS in both *w^1118^*-HFD flies and *w^1118^*-ND flies (both *P*<0.05), and it also increased heart diastolic diameters in both *w^1118^*-HFD flies and *w^1118^*-ND flies (both *P*<0.05). Importantly, there was no significant difference between *w^1118^*-HFD+E flies and *w^1118^*-ND flies in FS (*P*>0.05) (Fig. 2A–C). Moreover, a HFD significantly increased arrhythmia index (AI) in untrained *w^1118^* flies (*P*<0.05). Exercise reduced AI in *w^1118^*-HFD flies (*P*<0.05). There was no significant difference between *w^1118^*-HFD+E flies and *w^1118^*-ND flies in AI (*P*>0.05) (Fig. 2D). These results confirmed that a HFD could weaken heart contractility and increase the risk of arrhythmia in *w^1118^* flies, but endurance exercise could prevent this from happening in a HFD heart (Fig. 2E1–E4).

**Fig. 3.**
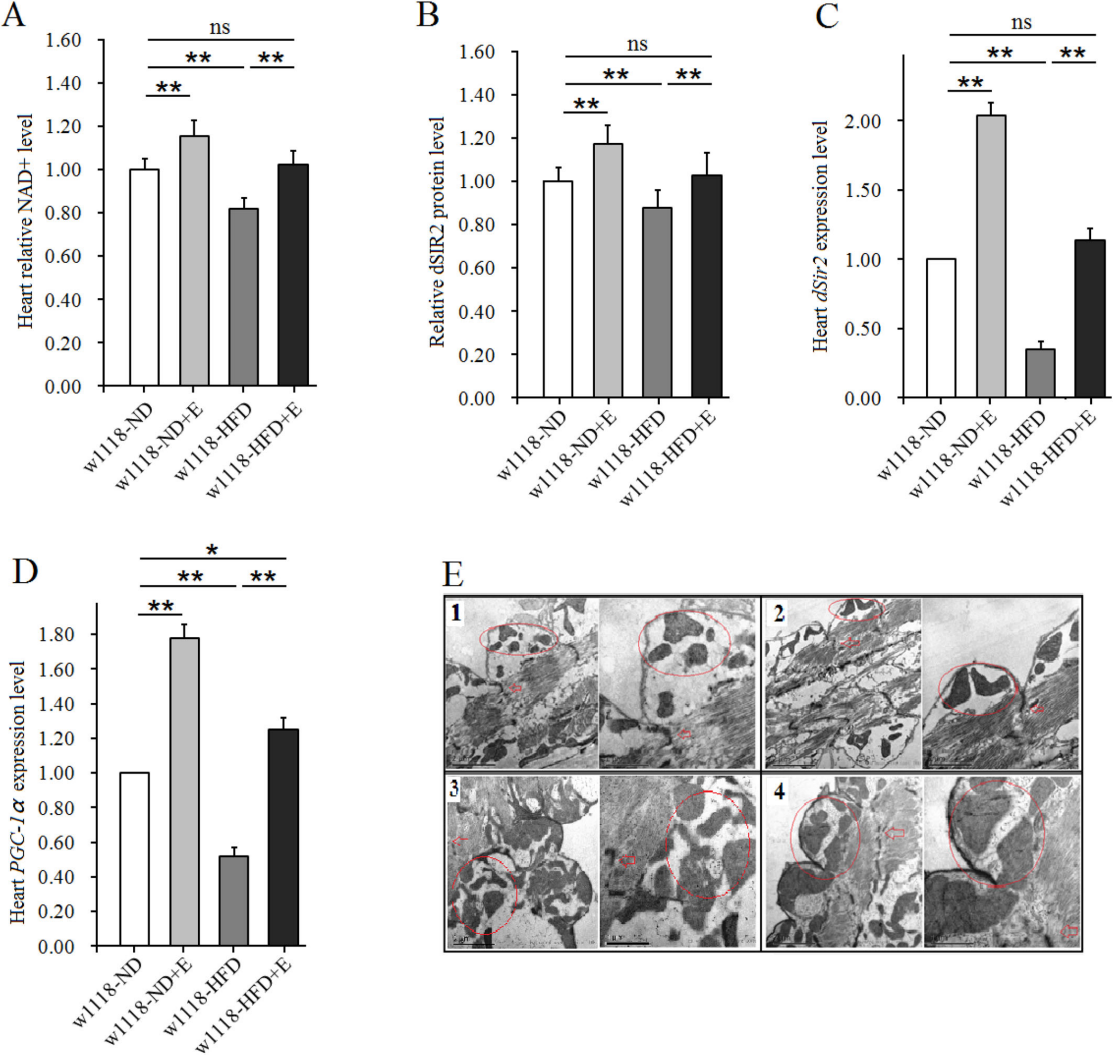
**The effect of a HFD and exercise on NAD^+^/dSIR2/*PGC-1α* pathway.** (A) Heart NAD^+^ levels. Results are expressed as the fold difference compared with *w^1118^*-ND flies. (B) Heart dSIR2 level. Results are expressed as the fold difference compared with *w^1118^*-ND flies. (C) Heart *dSir2* mRNA expression level. (D) Heart *PGC-1α* mRNA expression level. (E) The images of transmission electron microscopy; E1: *w^1118^*-ND; E2: *w^1118^*-ND+E; E3: *w^1118^*-HFD; E4: *w^1118^*-HFD+E. As we have seen exercise increased the mitochondrial numbers and improved myofibril arrangement regularity in myocardial cells. The red circle represented the position of the mitochondria in myocardial cells. The arrow represented the position of the Z line in myocardial cells. A two-way ANOVA was used to identify differences among the ND, ND+E, HFD, and HFD+E groups in *w^1118^* flies. Data are represented as means±s.e.m. *P<0.05; **P<0.01. The sample size of these indicators was 80 hearts, with three biological replicates.

The results also showed that a HFD significantly reduced cardiac NAD^+^ level, dSIR2 level, heart *dSir2* expression and *PGC-1α* expression level in untrained *w^1118^* flies (*P*<0.05, *P*<0.01). Exercise significantly increased cardiac NAD^+^ level, dSIR2 level, heart *dSir2* expression and *PGC-1α* expression level in both *w^1118^*-HFD flies and *w^1118^*-ND flies (*P*<0.01). Importantly, the cardiac *PGC-1α* expression levels in *w^1118^*-HFD+E flies was higher than that of *w^1118^*-ND flies (*P*<0.05) (Fig. 3A–D). Since the *PGC-1α* was involved in the synthesis of mitochondria, the number and morphology of mitochondria in cardiac cells was determined by transmission electron microscopy. We observed that in both HFD flies and non-HFD flies, exercise increased mitochondrial numbers and improved myofibril arrangement regularity in myocardial cells (Fig. 3E1–E4). Therefore, these results confirmed that a HFD induced a decreased in heart NAD^+^/dSIR2/*PGC-1α* pathway activity, but exercise training could prevent this from happening and even improve heart NAD^+^/dSIR2/*PGC-1α* pathway in HFD flies.

### Lipotoxic cardiomyopathy and cardiac *dSir2* gene in *Drosophila*

Sir2 is an archetypal longevity gene and founder of the Sirtuin protein family, and it is involved in the regulation of rDNA stability and the control of cellular lifespan and aging (Burnett et al., 2011; Kayashima et al., 2017; Slade and Staveley, 2016; Whitaker et al., 2013). Recent studies report that *dSir2* also takes part into the regulation of lipid metabolism (Hoffmann et al., 2013; Wen et al., 2018). For instance, overexpression of *dSir2* restricts fat accumulation in individual cells of the fat body in a cell autonomous manner, and loss of the *dSir2* leads to the age-progressive onset of hyperglycemia, obesity, glucose intolerance and insulin resistance (Palu and Thummel, 2016; Reis et al., 2010). However, it remains unclear how *dSir2* regulates lipid metabolism. Some evidence suggests that *Sir2* expression can alter the transcriptional activity of the mitochondrial biogenesis coactivator PGC-1α, and it catalyzes PGC-1α deacetylation both *in vitro* and *in vivo*. Overexpression of Sir2 deacetylase or increasing NAD^+^ levels activate transcriptional activity of *PGC-1α* in neurons and increases mitochondrial density (Dabrowska et al., 2016; Lan et al., 2017). It has been reported the *PGC-1α* in fly heart is a key gene in regulating the formation of lipotoxic cardiomyopathy (Diop et al., 2015; Dumont et al., 2018). To confirm the guess that heart *dSir2* gene can regulate cardiac lipid metabolism via modulating NAD^+^/dSIR2/*PGC-1α* pathway, the cardiac *dSir2* gene was overexpressed and knocked down by building UAS/hand-Gal4 system in *Drosophila*.

### Cardiac *dSir2* overexpression reduced the risk of lipotoxic cardiomyopathy

**Fig. 4.**
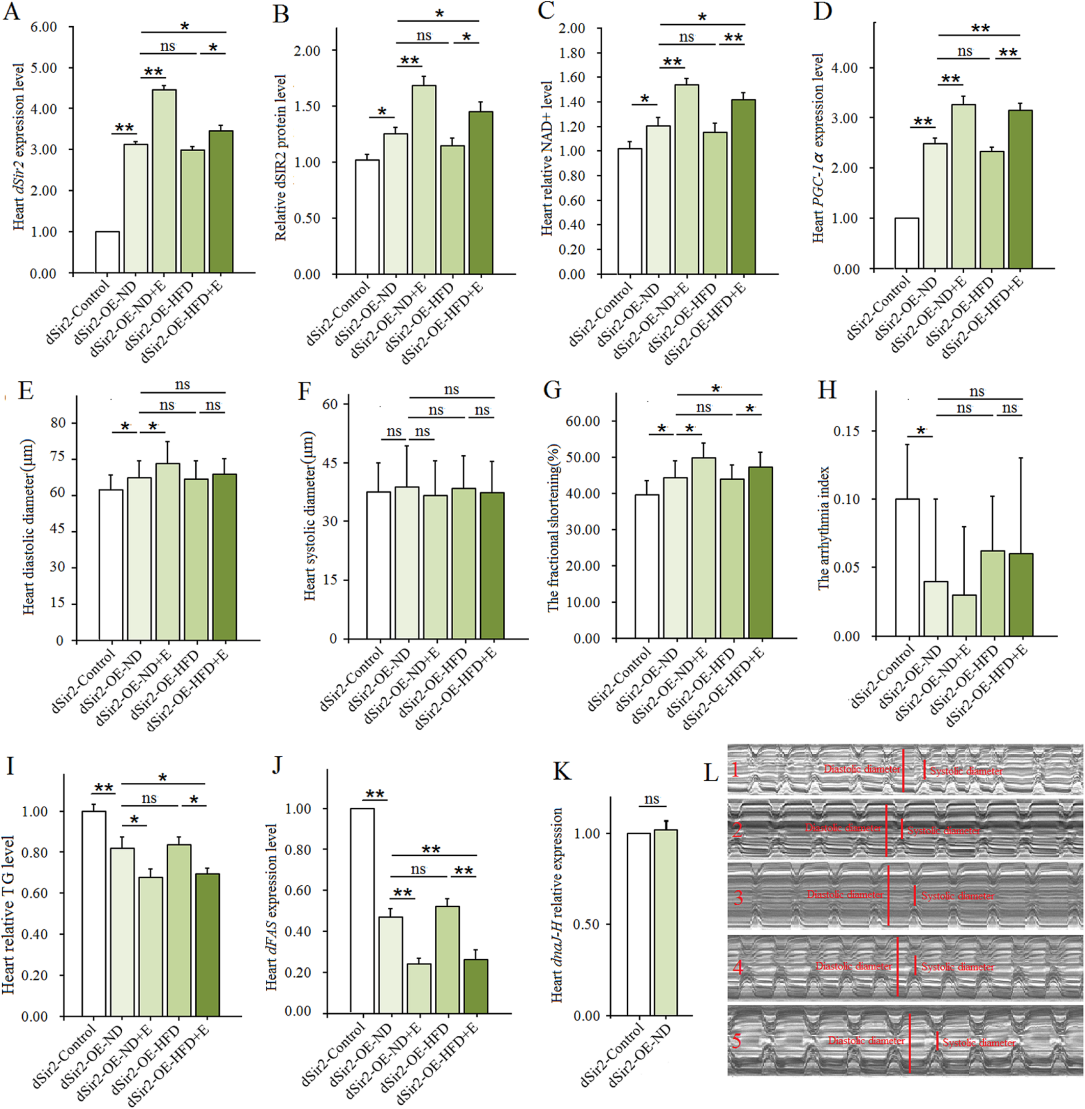
**The effect of a HFD and exercise on lipotoxic cardiomyopathy in *dSir2*-OE flies.** (A) Relative heart *dSir2* mRNA expression level. (B) Heart dSIR2 level. (C) Heart NAD^+^ levels. (D) Relative heart *PGC-1α* mRNA expression level. (E) Heart diastolic diameter. (F) Heart systolic diameter. (G) Fractional shortening. (H) Arrhythmia index. (I) Heart TG levels. Results are expressed as the fold difference compared with *dSir2-*control flies. (J) Relative heart *dFAS* gene expression level. (K) Relative heart *dnaJ-H* mRNA expression level. (L) Illustrating qualitative differences in heart function parameters (6 s): fractional shortening and arrhythmia index; L1: *dSir2*-control; L2: *dSir2*-OE-ND; L3: *dSir2*-OE-ND+E; L4: *dSir2*-OE-HFD; L5: *dSir2*-OE-HFD+E. Independent sample *t*-test was used to identify differences between *dSir2*-control flies and *dSir2*-OE flies. A two-way ANOVA was used to identify differences among the ND, ND+E, HFD, and HFD+E groups in *dSir2*-OE flies. Data are represented as means±s.e.m. *P<0.05; **P<0.01. The sample size was the same as with *w^1118^*.

In this research, the results showed the cardiac *dSir2* mRNA expression of *dSir2*-overexpression-normal diet (*dSir2*-OE-ND) flies was higher than that of *dSir2*-control flies (*P*<0.01, about 3.1-fold higher) (Fig. 4A). This suggested that cardiac *dSir2* gene overexpression was successfully constructed. Since a dnaJ-homologue (*dnaJ-H*) gene partially overlaps with *dSir2*, the cardiac *dSir2* overexpression might affect cardiac *dnaJ-H* mRNA expression. To avoid the influence of cardiac *dnaJ-H* mRNA expression on our results, we measured the expression of *dnaJ-H* gene in the heart*.* The results showed that there was no significant difference between *dSir2*-control flies and *dSir2*-OE flies in cardiac *dnaJ-H* mRNA expression (*P*>0.05) (Fig. 4K). Increasing evidence indicates that moderately increased expression of *dSir2* (2.5-fold, threefold, and fivefold) from the native *dSir2* locus cannot increase *dnaJ-H* mRNA expression (Burnett et al., 2011; Hoffmann et al., 2013). In this study, cardiac *dSir2* mRNA expression of *dSir2*-OE-ND flies was about 3.1-fold higher than that of *dSir2*-control flies. This hinted that cardiac *dSir2* overexpression did not significantly affect cardiac *dnaJ-H* mRNA expression, and cardiac *dnaJ-H* gene may not affect our results in this study. Also, results showed that cardiac *dSir2* overexpression significantly increased heart dSIR2 level, NAD^+^ level, and *PGC-1α* expression level (*P*<0.05, *P*<0.05, *P*<0.01) when *dSir2*-OE-ND flies were compared to *dSir2*-control flies (Fig. 4B–D). It hinted that cardiac *dSir2* gene overexpression upregulated NAD^+^/dSIR2/*PGC-1α* pathway activity. Moreover, we found heart diastolic diameter and fractional shortening of *dSir2*-OE-ND flies were higher than that of *dSir2*-control flies (*P*<0.05) (Fig. 4E,G). The arrhythmia index of *dSir2*-OE-ND flies was lower than that of *dSir2*-control flies (*P*<0.05) (Fig. 4H,L). This suggested that cardiac *dSir2* gene overexpression could enhance cardiac contractility and decrease the risk of arrhythmia. Finally, we found that the heart TG level and *dFAS* expression of *dSir2*-OE-ND flies was lower than that of *dSir2*-control flies (*P*<0.01) (Fig. 4-I,J). Therefore, these results indicated that cardiac *dSir2* gene overexpression could prevent lipid accumulation in the heart. According to others and our results, we hypothesized that cardiac *dSir2* gene overexpression could reduce the risk of lipotoxic cardiomyopathy via activating cardiac NAD^+^/dSIR2/ *PGC-1α* pathway in old flies.

To further confirm whether cardiac *dSir2* gene overexpression could prevent lipotoxic cardiomyopathy induced by a HFD, the *dSir2*-OE flies were fed a HFD. Results showed that the cardiac *dSir2* expression level, dSIR2 level, NAD^+^ level, *PGC-1α* expression level, diastolic diameter, fractional shortening, arrhythmia index, heart TG level and *dFAS* expression of *dSir2*-OE-ND flies were not significantly different from that of *dSir2*-OE-HFD flies (*P*>0.05) (Fig. 4). These results confirmed that cardiac *dSir2* gene overexpression could prevent lipotoxic cardiomyopathy induced by a HFD in old flies. The mechanism is that a HFD cannot weaken the activity of NAD^+^/dSIR2/*PGC-1α* pathway in *dSir2*-OE flies.

In *w^1118^* flies, we have confirmed that endurance exercise could resist lipotoxic cardiomyopathy and activate NAD^+^/dSIR2/*PGC-1α* pathway, but the relationship between endurance exercise and cardiac *dSir2* gene overexpression in preventing lipotoxic cardiomyopathy induced by a HFD remained unknown. So, the *dSir2*-OE flies participated in exercise training. We found that endurance exercise significantly upregulated the expression of cardiac *dSir2* gene in both *dSir2*-OE-ND flies and *dSir2*-OE-HFD flies (*P*<0.01 and *P*<0.05, respectively) (Fig. 4A), and it also remarkably increased heart dSIR2 level, NAD^+^ level and *PGC-1α* expression level in both *dSir2*-OE-ND flies and *dSir2*-OE-HFD flies (*P*<0.05 and *P*<0.01, respectively) (Fig. 4B–D). In addition, endurance exercise significantly increased diastolic diameter in *dSir2*-OE-ND flies (*P*<0.05), and it significantly increased fractional shortening in both *dSir2*-OE-ND flies and *dSir2*-OE-HFD flies (*P*<0.055) (Fig. 4E–G). Moreover, endurance exercise significantly reduced heart TG level and *dFAS* expression in both *dSir2*-OE-ND flies and *dSir2*-OE-HFD flies (*P*<0.05 and *P*<0.01, respectively) (Fig. 4I,J). Therefore, in the fight against lipotoxic cardiomyopathy, overexpression of cardiac *dSir2* was parallel to exercise training. Overexpression of cardiac *dSir2* combined with exercise training could better prevent lipotoxic cardiomyopathy in old flies. The mechanism was that both overexpression of cardiac *dSir2* and exercise training could superimpose the improvement of the activity of cardiac NAD^+^/dSIR2/*PGC-1α* pathway.

### Exercise improved lipotoxic cardiomyopathy induced by cardiac *dSir2* knockdown

**Fig. 5.**
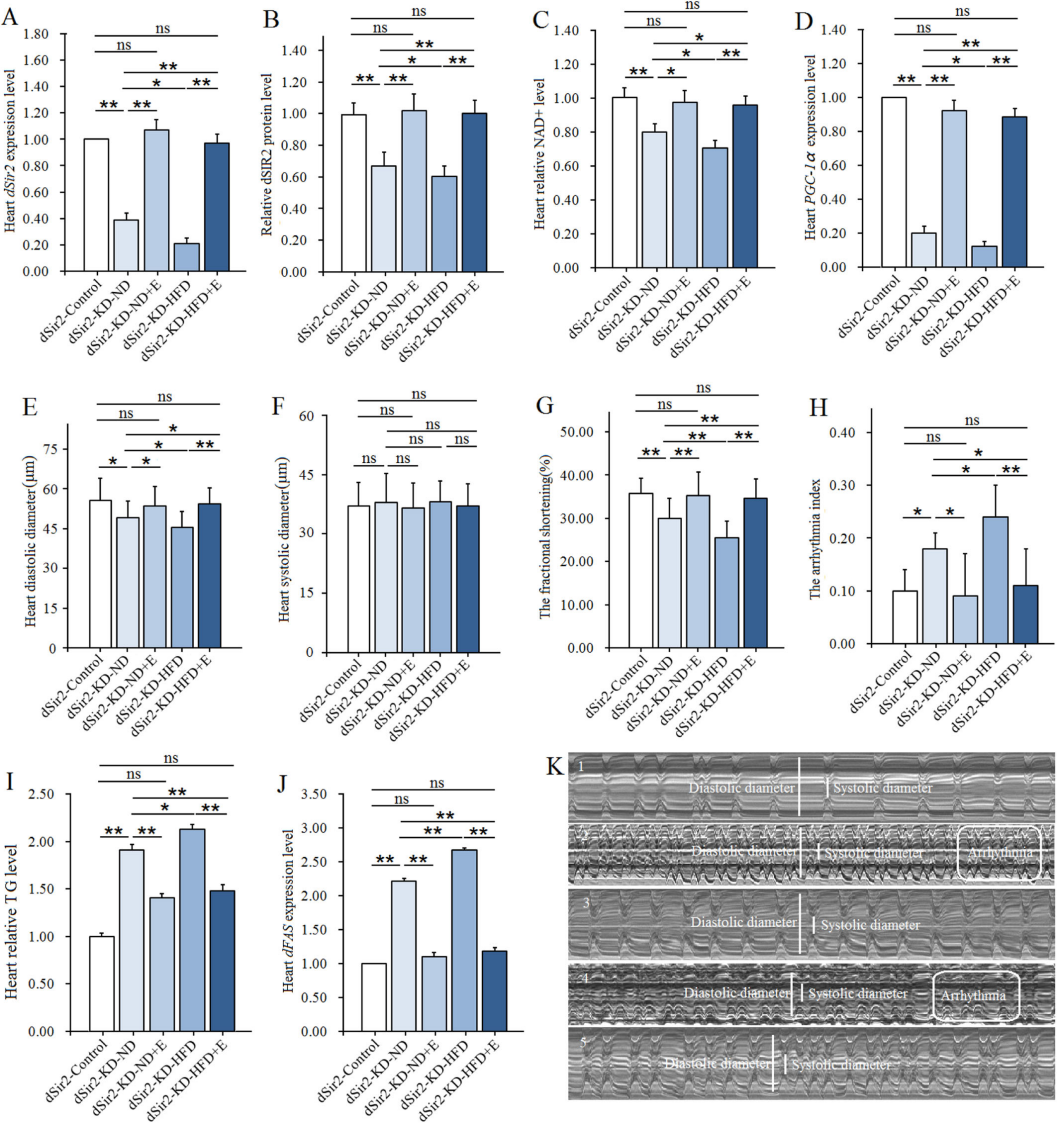
**The effect of a HFD and exercise on lipotoxic cardiomyopathy in *dSir2*-KD flies.** (A) Relative heart *dSir2* mRNA expression level. (B) Heart dSIR2 level. (C) Heart NAD^+^ levels. (D) Relative heart *PGC-1α* mRNA expression level. (E) Heart diastolic diameter. (F) Heart systolic diameter. (G) Fractional shortening. (H) Arrhythmia index. (I) Cardiac TG level. Results are expressed as the fold difference compared with *dSir2-*control flies. (J) Relative heart *dFAS* gene expression level. (K) Illustrating qualitative differences in heart function parameters (10 s): fractional shortening and arrhythmia index; K1: *dSir2*-control; K2: *dSir2*-KD-ND; K3: *dSir2*- KD-ND+E; K4: *dSir2*- KD-HFD; K5: *dSir2*-KD-HFD+E. Independent sample *t*-test was used to identify differences between *dSir2*-control flies and *dSir2*-KD flies. A two-way ANOVA was used to identify differences among the ND, ND+E, HFD, and HFD+E groups in *dSir2*-OE flies. Independent sample *t*-test was used to identify differences between *dSir2*-control flies and *dSir2*-KD flies. Data are represented as means±s.e.m. *P<0.05; **P<0.01. The sample size was the same as with *w^1118^*.

To further confirm the relationship between cardiac *dSir2* and lipotoxic cardiomyopathy, it was necessary to construct cardiac *dSir2* knockdown (KD) by UAS/hand-Gal4 system. In this study, the results showed that the cardiac *dSir2* mRNA expression of *dSir2*-KD-ND flies was lower than that of *dSir2*-control flies (*P*<0.01, about 2.5-fold lower) (Fig. 5A). It suggested that cardiac *dSir2* gene knockdown was successfully constructed. In addition, when *dSir2*-OE-ND flies were compared to *dSir2*-control flies, we found cardiac *dSir2* knockdown significantly decreased heart dSIR2 levels, NAD^+^ levels, and *PGC-1α* expression levels (*P*<0.01) (Fig. 5B–D). This suggested that cardiac *dSir2* gene knockdown inhibited NAD^+^/dSIR2/*PGC-1α* pathway activity. Moreover, we found the heart diastolic diameter and fractional shortening of *dSir2*-KD-ND flies were lower than that of *dSir2*-control flies (*P*<0.05, *P*<0.01) (Fig. 5E,G). The arrhythmia index of *dSir2*-KD-ND flies was higher than that of *dSir2*-control flies (*P*<0.05) (Fig. 5H,K). This suggested that cardiac *dSir2* gene knockdown could weaken cardiac contractility and increase the risk of arrhythmia. Finally, we found that the heart TG levels and *dFAS* expression of *dSir2*-KD-ND flies were higher than that of *dSir2*-control flies (*P*<0.01) (Fig. 5I,J). This indicated that cardiac *dSir2* gene knockdown could induce lipid accumulation in the heart. Therefore, according to others’ and our own evidence, we hypothesized that cardiac *dSir2* gene knockdown could induce lipotoxic cardiomyopathy via inhibiting cardiac NAD^+^/dSIR2/ *PGC-1α* pathway in old flies.

Although it had been confirmed that a HFD could induce lipotoxic cardiomyopathy in *w^1118^* flies, it remained unclear whether lipotoxic cardiomyopathy, induced by cardiac *dSir2* knockdown, could be aggravated after a HFD intervention. To figure this out, the cardiac *dSir2* gene knockdown flies were fed a HFD. Results showed that the cardiac *dSir2* expression level, dSIR2 level, NAD^+^ level and *PGC-1α* expression level of *dSir2*-KD-HFD flies were lower than that of *dSir2*-KD-ND flies (*P*<0.05) (Fig. 5A–D). This suggested that a HFD could reduce cardiac *dSir2* gene expression and the activity of NAD^+^/dSIR2/*PGC-1α* pathway in untrained *dSir2*-KD flies. In addition, heart diastolic diameter and fractional shortening of *dSir2*-KD-HFD flies were lower than that of *dSir2*-KD-ND flies (*P*<0.05, *P*<0.01) (Fig. 5E,G), and the arrhythmia of *dSir2*-KD-HFD flies was higher than that of *dSir2*-KD-ND flies (*P*<0.05) (Fig. 5H,K). It indicated that a HFD could weaken cardiac contractility and increase the risk of arrhythmia in untrained *dSir2*-KD flies. What is more, the heart TG level and *dFAS* expression of *dSir2*-KD-HFD flies were higher than that of *dSir2*-KD-ND flies (*P*<0.05, *P*<0.01) (Fig. 5I,J). It also indicated that a HFD could increase cardiac lipid accumulation in untrained *dSir2*-KD flies. So, these results confirmed that a HFD could aggravate lipotoxic cardiomyopathy induced by cardiac *dSir2* knockdown via inhibiting cardiac NAD^+^/dSIR2/*PGC-1α* pathway in untrained flies.

Although it had been identified that exercise could prevent lipotoxic cardiomyopathy induced by a HFD in *w^1118^* flies, and although exercise combined with overexpression of cardiac *dSir2* could better prevent lipotoxic cardiomyopathy in old flies, it remained unknown as to whether endurance exercise could improve lipotoxic cardiomyopathy induced by cardiac *dSir2* knockdown, and whether endurance exercise could prevent further deterioration of lipotoxic cardiomyopathy induced by a HFD in cardiac *dSir2* knockdown flies. To figure this out, the *dSir2*-KD-ND flies and *dSir2*-KD-HFD flies were given exercise training. Results showed that endurance exercise significantly upregulated the expression of cardiac *dSir2* gene in both *dSir2*-KD-ND flies and *dSir2*-KD-HFD flies (*P*<0.01) (Fig. 5A), and it also remarkably increased heart dSIR2 level, NAD^+^ level and *PGC-1α* expression level in both *dSir2*-KD-ND flies and *dSir2*-KD-HFD flies (*P*<0.05 and *P*<0.01, respectively) (Fig. 5B–D). In addition, endurance exercise significantly increased diastolic diameter and fractional shortening in both *dSir2*-KD-ND flies and *dSir2*-KD-HFD flies (*P*<0.05 and *P*<0.01, respectively) (Fig. 5E,G), and it significantly decreased arrhythmia index in both *dSir2*-KD-ND flies and *dSir2*-KD-HFD flies (*P*<0.05 and *P*<0.01, respectively) (Fig. 5H,K). Moreover, endurance exercise significantly reduced heart TG level and *dFAS* expression in both *dSir2*-KD-ND flies and *dSir2*-KD-HFD flies (*P*<0.01) (Fig. 5I,J). Therefore, we hypothesized that endurance exercise could improve lipotoxic cardiomyopathy induced by cardiac *dSir2* knockdown, and it could prevent further deterioration of lipotoxic cardiomyopathy induced by a HFD in cardiac *dSir2* knockdown flies.

However, to find out the extent to which exercise saved lipotoxic cardiomyopathy in both *dSir2*-KD-ND flies and *dSir2*-KD-HFD flies, the *dSir2*-KD-HFD+E flies were compared with *dSir2*-KD-ND flies, and the *dSir2*-KD-ND flies and *dSir2*-KD-HFD flies were compared with *dSir2*-control flies. Interestingly, the cardiac *dSir2* expression level, dSIR2 level, NAD^+^ level, *PGC-1α* expression level, diastolic diameter and fractional shortening of *dSir2*-KD-HFD+E flies were higher than that of *dSir2*-KD-ND flies (*P*<0.05 and *P*<0.01, respectively) (Fig. 5A–E,G). The arrhythmia index, heart TG level, and *dFAS* expression of *dSir2*-KD-HFD+E flies were lower than that of *dSir2*-KD-ND flies (*P*<0.05, *P*<0.01) (Fig. 5H–J). Moreover, the cardiac *dSir2* expression level, dSIR2 level, NAD^+^ level, *PGC-1α* expression level, diastolic diameter, fractional shortening, arrhythmia index, heart TG level and *dFAS* expression of *dSir2*-KD-ND flies and *dSir2*-KD-HFD flies were not significantly different from that of *dSir2*-KD-HFD flies (*P*>0.05) (Fig. 5). So, we confirmed that endurance exercise could resist and treat lipotoxic cardiomyopathy induced by a HFD and cardiac *dSir2* knockdown flies.

## DISCUSSION

### Lipotoxic cardiomyopathy induced by a HFD related to inhibiting NAD^+^/dSIR2/*PGC-1α* pathway

Increasing evidence showed that a HFD could induce lipotoxic cardiomyopathy, which manifested as cardiac lipid accumulation, reduced cardiac contractility, increased risk of arrhythmia and severe structural pathologies in flies (Birse et al., 2010; Diop et al., 2015; Na et al., 2013; Wen et al., 2018). Similarly, our results confirmed again that a HFD could induce lipotoxic cardiomyopathy. For example, a HFD increased the heart TG level and the heart *dFAS* expression levels, and it resulted in heavy lipid accumulation. In addition, a HFD reduced heart fractional shortening via decreasing diastolic diameter, and it increased arrhythmia indexes. These changes easily led to heart dysfunction and heart failure (Axell et al., 2015; Kang et al., 2016; Vaduganathan et al., 2018). Moreover, the heart NAD^+^ levels, dSIR2 levels, *dSir2* gene expression levels and *PGC-1α* expression were all decreased after feeding a HFD. These results suggested the heart NAD^+^/dSIR2/*PGC-1α* pathway activation was inhibited by a HFD.

Accumulating studies indicated that HFD-induced obesity could decrease the levels of NAD^+^ by several ways. For example, the mitochondria had compromised function due to overload that was thought to be induced by excessive beta-oxidation, and HFD-induced obesity could also decrease the mitochondrial numbers (Agil et al., 2015; Yoshino et al., 2011). Since there was a NAD^+^ pool in mitochondrial, NAD^+^ levels in metabolic tissues decreased with obesity (Alano et al., 2007; Di Lisa et al., 2001; Sauve, 2008). In addition, the oxidative stress by a HFD also caused an increased in lipid peroxidation, and the increased oxidative stress accumulated fat was an important pathogenic mechanism of metabolic syndromes associated with obesity (de Paula et al., 2016; Roberts et al., 2006). A number of studies had demonstrated that oxidative stress induced excessive PARP-1 activation mediates cell death, and NAD^+^ depletion mediates PARP-1-induced cell death, which indicated that HFD-induced increased oxidative stress could also reduce NAD^+^ level via PARP-1 activation mediated cell death (Horton et al., 2005; Konecny and Kristeleit, 2016; Massudi et al., 2012; Morales et al., 2014; Pang et al., 2015). NAD^+^ depletion lead to abnormal hepatic lipid metabolism in HFD-induced nonalcoholic fatty liver disease (Zhang et al., 2014). Therefore, these reports hinted that a HFD induced decreased NAD^+^ levels in cells.

In HFD flies, because of the lipotoxicity the reduction of cardiac NAD^+^ content inhibited dSIR2 (NAD-dependent histone deacetylases) activity. Since *PGC-1α* was a key antagonist of HFD-induced lipotoxic cardiomyopathy, and since PGC-1α was activated by the NAD^+^-dependent deacetylate of SIR2 (Arany et al., 2008; Rodgers et al., 2005), HFD-induced the reduction of NAD^+^ content decreased PGC-1α activation via inhibiting dSIR2 activation. This finally led to a more severe mitochondria number reduction, lipid accumulation, and dysfunction in the heart of HFD-fed flies. These findings suggested that the activity of NAD^+^/dSIR2/*PGC-1α* pathway was closely related to the formation of lipotoxic cardiomyopathy in flies. However, to confirm the relationship between NAD^+^/dSIR2/*PGC-1α* pathway and lipotoxic cardiomyopathy, further experiments should be done.

### Cardiac *dSir2* gene involved into regulating the formation of lipotoxic cardiomyopathy

It had been proposed that *Sir2* in the fat body plays an important role in regulating fat storage and mobilization, as *Sir2/Sirt1* had been implicated in regulation of fat metabolism in flies and mammals. Overexpression of *Sir2* in the adult fat body was found to be sufficient to extend the lifespan of male and female *Drosophila* (Boutant and Cantó, 2016; Hoffmann et al., 2013; Knight and Milner, 2012). However, it remains unclear whether *Sir2* can regulate heart lipid metabolism and cardiac function.

As expected, our research showed that the *dSir2* gene could also regulate lipid metabolism in the heart. For example, overexpression of cardiac *dSir2* gene could not only reduce heart TG accumulation and heart *dFAS* expression, but also it could prevent heart TG accumulation and decrease heart *dFAS* expression induced by a HFD. While cardiac *dSir2* knockdown could increase heart TG accumulation and heart *dFAS* expression, and this would get worse under a HFD intervention. The *dSir2* apparently regulates expression of genes involved in fat metabolism, and lack of *Sir2* increases fat deposition under normal conditions and consequently impairs starvation survival capabilities of flies (Banerjee et al., 2012). It has been reported that the FOXO and PGC-1*α* activity can be modulated by SIR2 deacetylation (Dabrowska et al., 2016; Lan et al., 2017), and these two factors are important to the formation of lipotoxic cardiomyopathy in *Drosophila* (Diop et al., 2015; Dumont et al., 2018). However, there is no direct evidence that cardiac *Sir2* can regulate heart lipid metabolism via activating PGC-1*α* activity in *Drosophila*.

In this study, we also found that cardiac *dSir2* gene had the ability to modulate cardiac function. For instance, overexpression of cardiac *dSir2* gene could increase heart fractional shortening via increasing diastolic diameter, and overexpression of cardiac *dSir2* gene could decrease arrhythmia index. In addition, overexpression of cardiac *dSir2* gene could prevent fractional shortening decline and arrhythmia index increase induced by a HFD. Also, cardiac *dSir2* knockdown could decrease heart fractional shortening via decreasing diastolic diameter, and cardiac *dSir2* knockdown could increase arrhythmia index. A HFD intervention could aggravate cardiac systolic dysfunction and arrhythmia in cardiac *dSir2* knockdown flies. It has been reported that *FOXO* or *PGC-1α* knockdown results in serious cardiac dysfunction in flies, including reduced cardiac contractility and increased the risk of arrhythmia (Diop et al., 2015; Dumont et al., 2018). The activity of PGC-1α can be modulated by SIR2 deacetylation (Dabrowska et al., 2016; Lan et al., 2017). However, there was no direct evidence that cardiac *dSir2* could regulate heart function via activating PGC-1*α* activity in *Drosophila*.

To confirm whether cardiac *dSir2* gene could regulate lipotoxic cardiomyopathy by modulating cardiac NAD^+^/ dSIR2/*PGC-1α* pathway, the cardiac dSIR2 level, NAD^+^ level and *PGC-1α* expression levels were measured. We found overexpression of cardiac *dSir2* gene increased cardiac dSIR2 level, NAD^+^ level, and *PGC-1α* expression level, and overexpression of cardiac *dSir2* gene could prevent the decline of cardiac dSIR2 level, NAD^+^ level, and *PGC-1α* expression level induced by a HFD. On the other hand, cardiac *dSir2* knockdown decreased cardiac dSIR2 level, NAD^+^ level, and *PGC-1α* expression level. A HFD intervention could aggravate the decline of cardiac dSIR2 level, NAD^+^ level, and *PGC-1α* expression level in cardiac *dSir2* knockdown flies.

SIR2 directly binds to PGC-1α and deacetylated it in 293T cells and PC12 cells (Li et al., 2016). SIR2 stimulates the ability of PGC-1α to coactivate hepatocyte nuclear factor 4α, thereby positively regulating gluconeogenic genes in response to pyruvate in hepatic cells. In the same cell type, SIR2 also enhances the ability of PGC-1α to inhibit glycolytic genes in response to pyruvate (Yang et al., 2014). Furthermore, SIR2 affects fatty acid oxidation in adipocytes and knockdown of PGC-1α cancels the effect of overexpression of SIR2 upon fatty acid oxidation. Thus the availability of other family members also contributes to the net effect of sirtuins upon PGC-1α (Tang, 2016). Systemic deletion of SIR2 in mice induces the development of dilated cardiomyopathy, which is accompanied by mitochondrial dysfunction. Overexpression of SIR2 in pancreatic β-cells enhances insulin secretion in response to glucose and improves glucose metabolism by increasing ATP production via suppression of uncoupling protein-2 expression (Cao et al., 2016; Luu et al., 2013).

In this research, we identified that cardiac *dSir2* gene could regulate *PGC-1α* expression via NAD^+^ and dSIR2 level. This possibly caused dysfunction of the heart's mitochondria, and this could induce lipid accumulation and reduced contractile ability of the heart, and eventually result in lipotoxic cardiomyopathy. A previous study has confirmed that when *PGC-1* was overexpressed in heart, lipotoxic cardiomyopathy was prevented after flies were fed a HFD (Diop et al., 2015). Similarly, overexpression of *dSir2* also resisted lipotoxic cardiomyopathy induced by a HFD in this study. Therefore, based on this evidence, we declared that the activation of cardiac NAD^+^/dSIR2/*PGC-1α* pathway was a key pathway that regulated the formation of lipotoxic cardiomyopathy.

### Exercise improved lipotoxic cardiomyopathy induced by a HFD and *dSir2* knockdown in old *Drosophila*

A lot of studies have confirmed that appropriate endurance exercise is a healthy and economical way to prevent and cure obesity, and endurance exercise is also considered a good way to improve heart functional in obese or old individuals (Stanley et al., 2019). For example, exercise training can strengthen the heart's ability to use fatty acids to provide energy by increasing the activity of related enzymes, which prevents lipid excessive accumulation in the heart (Wang and Xu, 2017). Furthermore, exercise training improves heart function such as cardiac contractibility and exercise reduces heart failure in obese individuals (Goit, 2017; Kwak, 2013; May et al., 2016; Voulgari et al., 2013). Finally, exercise increases muscle NAD^+^ levels and neuron NAD^+^ levels, and it activates transcriptional activity of *PGC-1α* and increases mitochondrial density (Dabrowska et al., 2016; Green et al., 1992; Koltai et al., 2010; Lan et al., 2017). However, it remains unclear whether exercise can prevent lipotoxic cardiomyopathy by activating cardiac NAD^+^/dSIR2/*PGC-1α* pathway.

In this research, we found that in HFD-fed flies, the heart TG level and the heart *dFAS* expression level were significantly decreased after exercise training. In addition, exercise training increased heart fractional shortening via increasing diastolic diameters, and exercise training decreased arrhythmia index in a HFD-fed flies and cardiac *dSir2* knockdown flies. Finally, the heart NAD^+^ levels, dSIR2 levels, *dSir2* gene expression levels and *PGC-1α* expression were all increased after exercise training in HFD-fed flies. This suggested that exercise training reduced lipid accumulation, improved heart function, activated NAD^+^/dSIR2/*PGC-1α* pathway and reduced the risk of arrhythmia, which prevented lipotoxic cardiomyopathy formation.

In HFD-fed flies, on one hand, exercise training can strengthen the heart's ability to use fatty acids to provide energy by increasing the activity of related enzymes, which prevents excessive lipid accumulation in the heart (Wang and Xu, 2017). On the other hand, because the dSIR2 protein plays a pivotal role in PGC-1α function via NAD-dependent deacetylation (Huang et al., 2015; Jünger et al., 2003; Kobayashi et al., 2005; Rizki et al., 2011), and because the *PGC-1α* is a key antagonist of HFD-induced lipotoxic cardiomyopathy (Birse et al., 2010; Haemmerle et al., 2011; Puigserver et al., 1998; Wessells et al., 2004), exercise training increased the *PGC-1α* function via improving heart NAD^+^ content and heart dSIR2 activation in this study (Dabrowska et al., 2016; Green et al., 1992; Koltai et al., 2010; Lan et al., 2017). Therefore, these results suggested that the NAD^+^/dSIR2/*PGC-1α* pathway activation was an important molecular mechanism of exercise resistance against lipotoxic cardiomyopathy. However, it remained unclear whether exercise training could improve lipotoxic cardiomyopathy induced by cardiac *dSir2* knockdown.

In this study, the heart *dSir2*-RNAi flies were exercise trained and we found exercise training also reduced lipid accumulation, enhanced heart function, activated NAD^+^/dSIR2/*PGC-1α* pathway, and reduced the risk of arrhythmia, which improved lipotoxic cardiomyopathy induced by heart *dSir2* RNAi. In heart *dSir2*-RNAi flies, exercise training can also increase the cardiac ability to use fatty acids to provide energy by increasing the activity of related enzymes, which prevents lipid excessive accumulation in the heart (Wang and Xu, 2017). In addition, increasing evidence hints that exercise training can upregulate *dSir2* activity. For instance, it has been reported that exercise not only improves blood NAD^+^ levels but also in muscle and cardiac NAD^+^ levels (Cantó et al., 2010; Fukuwatari et al., 2001; Touati et al., 2015), which may eventually result in increasing NAD^+^ activity in these tissues and organs to meet the demand of NAD^+^ metabolism during exercise training. Since the *dSir2* activity can be regulated by free NAD^+^ in cells, the *dSir2* expression may be indirectly elevated by exercise trained (Gambini et al., 2011). Moreover, increasing evidence indicates that exercise can intensify the contraction of cardiac muscles which may facilitate SIRT1 upregulation. Exercise training can upregulate heart AMPK expression – AMPK is an energy sensor. Since AMPK also increases the intracellular NAD^+^ levels, its activity is correlated with SIRT1 enhancement (Chen et al., 2018; Lavu et al., 2008). Next, recent studies report that exercise training upregulates SIRT1 in kidney, liver and brain (Huang et al., 2019; Liu et al., 2019). So, these reports suggest that exercise training can increase *Sir2* expression. However, there is no evidence that exercise training can increase heart *dSir2* expression by affecting the UAS/Gal4 system. Besides, in our experiment, a stronger cardiac *dSir2* knockdown has not been generated any other way, such as with an inducible CRISPR or a stronger RNAi line or stronger driver. Therefore, our results indicated that exercise training rescued the cardiac *dSir2* expression and dSir2 protein levels only under this mild *dSir2*-knockdown condition, and the reason may be that exercise induction of cardiac *dSir2* was stronger than knockdown.

In conclusion, we identified that the heart *dSir2* gene and *dSir2*/NAD^+^/*PGC-1α* pathway regulated the heart lipid metabolism and the formation of lipotoxic cardiomyopathy. Lipotoxic cardiomyopathy could be induced by heart *dSir2* knockdown, but the heart *dSir2* overexpression could prevent a HFD-induced lipotoxic cardiomyopathy. Exercise training could improve lipotoxic cardiomyopathy induced by a HFD or heart *dSir2* knockdown in old *Drosophila*. The NAD^+^/dSIR2/*PGC-1α* pathway activation was an important molecular mechanism of exercise resistance against lipotoxic cardiomyopathy.

## MATERIALS AND METHODS

### Fly stocks, diet and husbandry

The *w^1118^* and *hand-Gal4* line was a gift from Xiu-shan Wu (Heart Development Center of Hunan Normal University). UAS-*dSir2*-OE (*w^1118^;P{EP}Sirt1^EP2300^DnaJ-H^EP2300^/CyO*) line was obtained from the Bloomington Stock Center, and the P{EP} construct carries Scer\UAS binding sites for the Scer\GAL4 transcriptional regulator, and bacterial sequences that allow plasmid rescue. The Gal4-UAS system allows regulated expression of genes proximate to the site of the insertion: genes properly oriented with respect to the Scer\UAS sequences can be conditionally expressed via transgene-derived Scer\GAL4 activity (Rorth, 1996). UAS-*dSir2*-KD (*w^1118^; P{GD11580}v23201*) line was obtained from the Vienna Drosophila RNAi Center. Male *hand-Gal4* flies were crossed to female UAS-*dSir2*-OE flies and UAS-*dSir2*-KD flies.

To avoid the influence of genetic background differences on the results, maternal origin was used as the genetic control. The female ‘*w^1118^;P{EP}Sirt1^EP2300^DnaJ-H^EP2300^/CyO*’ and ‘*hand-Gal4>w^1118^;P{EP}Sirt1^EP2300^DnaJ-H^EP2300^/CyO*’ were represented as ‘*dSir2*-control’ and ‘*dSir2*-OE’. The female ‘*w^1118^; P{GD11580} v23201*’ and ‘*hand-Gal4>w^1118^; P{GD11580} v23201*’ were represented as ‘*dSir2*-control’ and ‘*dSir2*-KD’. The female ‘*w^1118^*’, ‘*dSir2*-OE’, and ‘*dSir2*-KD’ virgin flies were divided into several groups: normal-diet (ND) group, normal-diet +exercise (ND+E) group, high-fat diet (HFD) group, and HFD+exercise (HFD+E) group.

Normal food contained 10% yeast, 10% sucrose and 2% agar. The HFD was made by mixing 30% coconut oil with the food in a weight to volume ratio with the normal food (Birse et al., 2010). Both HFD+E group flies and HFD-E group flies were fed the HFD from 28 days of age and were exposed to the HFD for 5 consecutive days. During the experimental time course, flies were housed in a 22±1°C incubator with 50% humidity and a 12-h light/dark cycle. This environment could keep the coconut oil food in a solid state since the melting point of coconut oil is about 24°C, thus ensuring that flies would not get stuck in the oily food. Fresh food was provided every other day for the duration of the experiment. All group flies were raised to the fourth weekend. Flies were trained or fed a HFD at 5 weeks old as we found flies were very sensitive to exercise or HFDs at this time.

### Exercise training device and protocols

The advantage of the flies' natural negative geotaxis behavior was taken to induce upward walking when constructing the exercise device (Tinkerhess et al., 2012). All exercise group flies started exercise from when they were 5 weeks old, and underwent a 5-day-long exercise program. Vials were loaded horizontally into a steel tube that was rotated about its horizontal axis by an electric motor, with a gear regulating its shaft speed. There were 25 flies in each vial. Thus, each vial was rotated along its long axis with the accompanying rotating steel tube, which made the flies climb. Most flies continued to respond by climbing throughout the exercise period. The few that failed to climb were actively walking at the inner wall of the vial (Wen et al., 2016; Zheng et al., 2015). Flies were exercised in vials with a 2.8-cm inner diameter, rotated at 0.14 rev/s. Flies were exercised for 1.5 hours every time.

### Semi-intact *Drosophila* preparation and image analysis

First, 30 flies were anesthetized with FlyNap for 2–3 min (a few flies were anesthetized with FlyNap for 4–5 min as they were hard to narcotize). Next, the head, ventral thorax and ventral abdominal cuticle were removed by special scissors and tweezers to expose the heart in the field of vision of a microscope. Note, dissections were done under oxygenated artificial hemolymph. These semi-intact preparations were allowed to equilibrate with oxygenation for 15–20 min before filming. Finally, image analysis of heart contractions was performed using high-speed videos of the preparations. Videos were taken 120–130 frames per second using a Hamamatsu EM-CCD digital camera on a Leica DM LFSA microscope with a 10 immersion lens. To get a random sampling of heart function, a single 30-s recording was made for each fly. All images were acquired and contrast enhanced by using Simple PCI imaging software. The heart physiology of the flies was assessed using a semi-automated optical heartbeat analysis program that quantifies heart diastolic diameters, systolic diameters, fractional shortening and arrhythmia index (Fink et al., 2009).

### The SIRT1/dSIR2 assay, NAD^+^ assay, and TG assay

For dSIR2 assay, 80 hearts were homogenized in 200 μl PBS buffer. To break the cells, hearts were subjected to freeze-thaw cycles. The homogenates were then centrifuged for 5 min at 5000×***g*** to get the supernate as a sample. We used purified insect SIRT1 antibody to coat microtiter plate wells, made the solid-phase antibody, then added SIRT1 to the wells, which, combined with HRP-labelled antibody, become antibody-antigen-enzyme-antibody complex. Then we added 50 μl standard sample liquid and experimental sample liquid to different wells. 100μl of HRP-conjugate reagent was added to each well, which were then incubated for 60 min at 37°C. 50 μl Chromogen Solution A and Chromogen Solution B were added to each well, which were kept in the dark for 15 min at 37°C. Next 50 μl of Stop Solution was added to each well to stop the reaction (a color change from blue to yellow was observed). The blank well was taken as zero, absorbance was read at 450 nm after adding Stop Solution and within 15 min. The OD values were used to determine sample Sirt1 concentration from the standard curve according to the manufacturer's instructions (Insect SIRT1 ELISA Kit, MLBIO).

For NAD^+^ assay, 80 hearts were homogenized in 200 μl NAD exaction buffer, and then extracts were heated at 70°C for 5 min. 20 μl assay buffer and 100 μl of the opposite extraction to neutralize the extracts were added. Samples were centrifuged at 14,000 rpm for 5 min and then 40 μl standard sample liquid and experimental sample liquid were transferred to separate wells. 80 μl of working reagent (40 μl assay buffer, 1 μl oxidation-reduction enzyme, 10 μl 10% ethanol, 20 μl PMS, 20 μl MTT) was quickly added to each well. The resulting samples were then measured at 565 nm with a microplate spectrophotometer. The OD values were used to determine sample NAD^+^ concentration from the standard curve according to the manufacturer's instructions (EnzyChrom™NAD^+^/NADH assay kit).

Cardiac TG measurements were taken from the hearts of 20 female flies dissected in artificial hemolymph. Care was taken to remove as much adipose tissue and other heart-associated cells from the heart as possible. Preparations were washed two times with PBS. Using fine forceps, the hearts were pulled off from the cuticle and transferred into Eppendorf tubes containing 26 μl of PBST (PBS+0.05% Triton X-100). Tubes were immediately frozen and stored at −80°C. Hearts were then mildly sonicated to lyse the cells. 20 μl heart lysates was transferred to a 96-well plate containing 200 μl of lipid reagent. After incubating 10′ at 37°C, 3 μl of heart lysate was transferred to wells containing 200 μl of Bradford reagent to measure protein content. The reaction mixture was incubated in an Environ Shaker at 300 rpm at 37°C for 10 min; the OD 550 nm was measured using SpectraMax Molecular Devices Corp and compared with a standardized curve. Experiments were repeated at least three times in multiple replicates.

### qRT-PCR

To check the transcriptional expression of the *dSir2*, *dFAS* and *PGC-1α* gene, 80 flies' hearts were homogenized in Trizol for each group. Firstly, 10 μg of the total RNA was purified by organic solvent extraction from the Trizol (TRIzol, Invitrogen). Next, the purified RNA was treated with DNase I (RNase-free, Roche) and used to produce oligo dT-primed cDNAs (SuperScript II RT, Invitrogen), which were then used as templates for quantitative real-time PCR. The rp49 gene was used as an internal reference for normalizing the quantity of total RNAs. Finally, Real-time PCR was performed with SYBR green using an ABI7300 Real time PCR Instrument (Applied Biosystems). Expression of the various genes was determined by the comparative CT method (ABI Prism 7700 Sequence Detection System User Bulletin #2, Applied Biosystems). Primer sequences of *dSir2* were as follows: F: 5′-GCAGTGCCAGCCCAATAA-3′; R: 5′-AGCCGATCACGATCAGTAGA-3′. Primer sequences of *PGC-1α* were as follows: F: 5′-TGTTGCTG CTACTGCTGCTT-3′; R: 5′-GCCTCTGCATCACCTACA CA-3′. Primer sequences of *dFAS* were as follows: F: 5′-GGTGAGACCATCGTGGAAGT-3′; R: 5′-AATGTCTGCCAAGCCAGAGT-3′. Primer sequences of *dnaJ-H* were as follows: F: 5′-GCAAGATGGCACACGTAGCTG-3′; R: 5′-CCACTGTAGCAAC ACGTAATCACC-3′. Primer sequences of Internal were as follows: F: 5′-CTAAGCTGTC GCACAAATGG-3′; R: 5′-AA CTTCTTG AATCCGGTGGG-3′.

### Statistical analyses

A two-way ANOVA was used to identify differences among the ND, ND+E, HFD, and HFD+E groups of *Drosophila* with the same genetic background. Independent sample *t*-test was used to identify differences between *dSir2*-control flies and *dSir2*-OE flies. Analyses were performed using the Statistical Package for the Social Sciences (SPSS) version 16.0 for Windows (SPSS Inc., Chicago, USA), with statistical significance set at *P*<0.05. Data are represented as means±s.e.m.
